# Personal innovative approach in radiation therapy of lung cancer- functional lung avoidance SPECT-guided (ASPECT) radiation therapy: a study protocol for phase II randomised double-blind clinical trial

**DOI:** 10.1186/s12885-021-08663-1

**Published:** 2021-08-21

**Authors:** Azza Ahmed Khalil, Eric Hau, Val Gebski, Cai Grau, Harriet Gee, Tine Bisballe Nyeng, Katrina West, Stine Kramer, David Farlow, Marianne Knap, Ditte Sloth Møller, Lone Hoffmann, Katherina P. Farr

**Affiliations:** 1 Aarhus University Hospital, Aarhus, Denmark; 2 Department of Radiation Oncology, Crown Princess Mary Cancer Centre, Westmead Hospital, Sydney West Radiation Oncology Network, Westmead, Australia; 3 Westmead Clinical School, University of Sydney, Sydney, NSW Australia; 4 Westmead Institute for Medical Research, Westmead, NSW Australia; 5 NHMRC Clinical Trials Centre, University of Sydney, Camperdown, 2050 Australia; 6 Westmead Hospital, Westmead, NSW Australia; 7 Faculty of Medicine, University of New South Wales, Sydney, NSW Australia

**Keywords:** Lung cancer, Radiation therapy, Radiation-induced lung toxicity, Functional imaging, Perfusion SPECT/CT

## Abstract

**Background:**

Radiation therapy (RT) plays a key role in curative-intent treatment for locally advanced lung cancer. Radiation induced pulmonary toxicity can be significant for some patients and becomes a limiting factor for radiation dose, suitability for treatment, as well as post treatment quality of life and suitability for the newly introduced adjuvant immunotherapy. Modern RT techniques aim to minimise the radiation dose to the lungs, without accounting for regional distribution of lung function. Many lung cancer patients have significant regional differences in pulmonary function due to smoking and chronic lung co-morbidity. Even though reduction of dose to functional lung has shown to be feasible, the method of preferential functional lung avoidance has not been investigated in a randomised clinical trial.

**Methods:**

In this study, single photon emission computed tomography (SPECT/CT) imaging technique is used for functional lung definition, in conjunction with advanced radiation dose delivery method in randomised, double-blind trial. The study aims to assess the impact of functional lung avoidance technique on pulmonary toxicity and quality of life in patients receiving chemo-RT for lung cancer. Eligibility criteria are biopsy verified lung cancer, scheduled to receive (chemo)-RT with curative intent. Every patient will undergo a pre-treatment perfusion SPECT/CT to identify functional lung. At radiation dose planning, two plans will be produced for all patients on trial. Standard reference plan, without the use of SPECT imaging data, and functional avoidance plan, will be optimised to reduce the dose to functional lung within the predefined constraints. Both plans will be clinically approved. Patients will then be randomised in a 2:1 ratio to be treated according to either the functional avoidance or the standard plan. This study aims to accrue a total of 200 patients within 3 years. The primary endpoint is symptomatic radiation-induced lung toxicity, measured serially 1–12 months after RT. Secondary endpoints include: a quality of life and patient reported lung symptoms assessment, overall survival, progression-free survival, and loco-regional disease control.

**Discussion:**

ASPECT trial will investigate functional avoidance method of radiation delivery in clinical practice, and will establish toxicity outcomes for patients with lung cancer undergoing curative chemo-RT.

**Trial registration:**

Clinicaltrials.gov Identifier: NCT04676828. Registered 1 December 2020.

**Supplementary Information:**

The online version contains supplementary material available at 10.1186/s12885-021-08663-1.

## Background

Lung cancer is the most commonly diagnosed cancer worldwide and one of the leading causes of cancer death with very poor prognosis [1]. Concurrent chemo-radiation therapy (RT) plays a significant role in the treatment, as up to 77% of all lung cancer patients may require RT at some point during the treatment [1]. Despite that, many patients do not receive this treatment due to concerns about radiation-induced lung toxicity (RILT). Up to 50% of patients may develop clinical signs of RILT weeks and months after RT, such as dyspnea, cough, chest pain and fever [2]. These symptoms may become chronic and reduce the patient’s reserve to deal with future cardiopulmonary stresses and receive anti-cancer therapy in the future [2, 3]. The condition may become life-threatening, whereas the development of RILT in some cases has shown to negatively affect patient’s survival [4]. Furthermore, the addition of 1 year of maintenance anti-PD-L1 therapy after definitive chemo-RT has become a new standard-of-care for patients with stage III non-small-cell lung cancer. In phase III PACIFIC clinical trial maintenance durvalumab have shown clinical benefits, but also potential for increased toxicity [5]. Given the morbidity associated with RILT and its increasing relevance in lung cancer, our ability to identify patients at risk for developing treatment related toxicity is crucial. Therefore, RT is a trade-off between eradicating the tumour and minimizing damage to the lung. The ability to avoid well-functioning lung tissue around the tumour points to a significant gap in our current treatment paradigm. An important assumption in RT is that functional activity is distributed homogeneously throughout the lungs. However, a significant proportion of lung cancer patients have defects in regional lung function caused by tumour, chronic lung disease and smoking [6]. For these patients undergoing potentially toxic therapy, pre-treatment assessment of lung function is very important. Single photon emission computed tomography (SPECT) is a well-established functional modality for diagnosis and monitoring of lung disease [7]. SPECT uses radioactive labelled tracer for imaging pulmonary circulation, where perfused areas equate with normal functional lung. Fused hybrid SPECT/CT images lung anatomy and function combined.

Over the last decade, several studies demonstrated that SPECT may be useful to guide radiotherapy to reduce RILT [8]. We have previously established the role of SPECT/CT in imaging of functional heterogeneity, prediction the risk of RILT, and showed dose-response relation [4, 6, 9]. The rationale of the functional lung avoidance approach is to guide the radiation away from functional lung regions. Thus, numerous studies demonstrated that incorporating functional imaging into treatment planning is feasible [8–12]. However, it remains to be proven clinically that functional image guided RT improves the toxicity outcomes of lung cancer patients. There is, therefore, a need for prospective interventional trials with clinical endpoints of radiation-induced toxicity to establish the role of functional lung avoidance RT in clinical practice. The ASPECT trial will implement functional imaging into radiotherapy of lung cancer to provide evidence for the effect of functional lung avoidance RT on clinical toxicity outcomes, quality of life, disease control and survival parameters.

## Methods/design

### General objective

To determine if SPECT-Perfusion Functional Avoidance improves toxicity outcomes for patients with lung cancer undergoing curative chemo-RT.

Endpoints.

The primary endpoint is symptomatic RILT, defined as number of patients developing pulmonary toxicity ≥ grade 2 in both treatment arms

- Measured using the National Cancer Institute Common Toxicity Criteria (NCI-CTC) version 5 for radiation pneumonitis, dyspnea, cough, or any other radiation-induced respiratory, thoracic and mediastinal disorder. Measured serially from 1 to 12 months after RT

Secondary endpoints are:

- Change in quality of life and patient reported lung symptoms according to the EORTC quality of life questionnaires QLQ-C30 and QLQ-LC-13. Measured serially from 1 to 12 months after RT.
- Dose-volume histogram parameters for lung and functional lung
- Progression-free survival, defined as time from randomisation to disease progression at any site or death
- Overall survival, defined as time from randomisation to death of any cause or last date known alive
- Loco-regional control rate at 12 months, defined as freedom from local disease progression 12 months after randomisation.

### Characteristic of the participants

- Histologically verified lung cancer (small-cell and non-small-cell lung cancer)
- Referred for RT with curative intent
- Radiation dose of 50–66 Gy given in 2-Gy fractions, other dose levels and fractionation schedules accepted, as per site standard
- Concurrent chemotherapy is accepted
- Concurrent immunotherapy is not allowed
- Previous RT to the thorax region is not allowed
- Patients with oligometastatic disease are allowed, where metastasis have been ablated with surgery or RT, receiving (chemo)-RT to the thoracic disease with curative intent
- Absence of other uncontrolled malignancies
- Absence of any psychological, familial, sociological, or geographical condition potentially hampering compliance with the study protocol
- Adults over 18, that have given oral and written informed consent before patient registration

### Study design

**Fig. 1:**
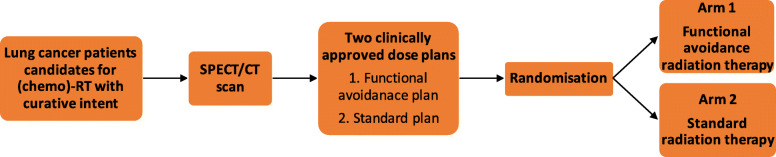
ASPECT study design

This study is a multicentre non-comparative double-blinded randomised phase II trial (Fig. 1). Participation implies baseline and follow-up procedures as indicated in Table 1. SPECT/CT will be performed on all patients included in the trial prior to randomisation. In the treatment planning phase, a standard and a functional avoidance plans will be produced for each patient. The patients will be randomised to one of the following arms: Arm 1- Functional avoidance radiation therapy and Arm 2- Standard radiation therapy.

**Table 1:** Summary of baseline and follow-up investigations

Required Investigations	Baseline	1 month	3 months	6 months	9 months	12 months
Subjective						
QLQ-C30 + LC13	X	X	X	X	X	X
Objective						
Physical examination	X	X	X	X	X	X
Toxicity scoring (CTCAE v5)	X	X	X	X	X	X
Management						
Medication	X	X	X	X	X	X
Analytic						
Blood samples	X		X	X		X
Lung function tests PFT, DLCO	X			X		X
Progress CT evaluation	X		X	X	X	X
SPECT/CT	X					
Adverse Events	X	X	X	X	X	X

Patient recruitment and data collection will take place at academic and regional Hospitals with expected 3–5 study sites in Denmark and Australia.

### Study procedures

#### SPECT/CT scan

In the lung, perfusion SPECT is able to image functioning pulmonary vasculature. SPECT perfusion imaging is acquired using a Siemens dual head SPECT-CT gamma camera (Symbia or Intevo), following intravenous injection of approximately 200 MBq of 99mTc - macro-aggregated albumin with the patient supine, typically in an arms-up position. A “step and shoot” methodology is used for data acquisition, with high-resolution collimation, 32 steps per head at 20 s per step. Low dose CT is concurrently acquired, without breath-hold, using parameters of 110 kV and 50mAs. Processing is via an ordered subset technique, using 6 subsets and 4 iterations with attenuation correction using CT data, as well as scatter and resolution recovery techniques, for more quantitatively robust MAA SPECT data. Patient positioning and fixation is done according to the standard procedures of the Nuclear Medicine Department. Total functional lung (FL) (all voxels with SPECT signal in the lungs excluding gross tumour volume (GTV)) is contoured. In addition, contours of functional lung are created from the SPECT signal using a threshold of 20–80% of maximum perfusion count (FLx, x = 20, 40, 60, 80%) for each patient individually, as described previously [9, 12]. These contours are transferred from the SPECT/CT to the planning CT using rigid registration and cropped to the delineated total lung volume.

#### Radiation therapy

Recommendations for radiation treatment of lung cancer developed by the Danish Oncologic Lung Cancer Group (DOLG) will be applied and used for planning scan, fixation technique, planning algorithm, target definition and coverage, image guidance, fractionation, organs at risk delineation and dose constraints [1]. Local treatment centres and research protocols guidelines may be applied. Radiotherapy regimen involves Positron emission tomography (PET/CT)-based dose planning. Radiation doses are delivered to the target with a minimization of associated irradiation of surrounding normal tissue. All patients, regardless participation in the trial will be treated with a prescribed dose to the target.

#### Patient data acquisition and patient positioning

Patients undergo free breathing PET/4DCT scan in the supine position (helical CT scanner with minimum 3 mm slice spacing). The patients are reproducibly fixated and immobilized. The scan is performed after injection of intravenous contrast, and the scan range covers the complete thoracic region.

#### Target delineation

All CT scans are transferred to the treatment planning system and GTV of the primary tumour and involved nodes as defined by FDG uptake on PET scan or positive histology from biopsy. GTV is expanded to a clinical target volume (CTV) by adding isotropic margins for primary tumour and for nodes. CTV can be modified in areas of overlap with large vessels, bones, trachea, chest wall and lung tissue without compromising the GTV – though never if the GTV extends into the adjacent tissue. By adding margins to the CTV to generate a planning target volume (PTV), it is ensured that the CTV is covered by the planned dose. These margins take into account the uncertainties associated with planning and treatment delivery. Margins must be supported by the image-guidance recommendations from DOLG guidelines. Respiratory motion can be included either in the delineation of the GTV or as a patient specific part of the PTV margin.

#### Organs at risk (OAR) delineation and constraints

The organs at risk are: lung parenchyma, esophagus, spinal cord, heart and body. Dose constraints to organs at risk are respected to minimize the risk of complications. The constraints on the spinal cord, heart and the volume of the lung receiving at least 20 Gy are following (unless otherwise specified for patients included in other protocols)

- Both lungs should be contoured in their entirety. The mean lung dose (MLD), the mean dose delivered to both lungs minus all GTV’s) will be calculated from dose-volume histogram (DVH) and should not exceed 20 Gy. The V_lung_20 (the volume of lung outside the tumour receiving a dose of more than 20Gy) should not exceed 35%, aim for V5 < 60%;
- The esophagus volume (outer muscular contour) must be contoured from the level of just below the larynx to the gastro-esophageal juncture. The PTV should not be subtracted from the total volume of the esophagus. Aim for dose to 1 cm^3^ under 100% of prescribed dose;
- For spinal cord dose evaluation, it is mandatory to contour the spinal cord. The calculated near maximum dose to the spinal cord should not exceed 45 Gy or max 50 Gy for delineated spinal canal;
- The heart dose should be kept as low as possible, following the constraints of V40 < 30% and V25 < 50%;
- Body: global hotspot< 112%.

#### Dose specification

After contouring the target and normal structures, photon treatment plans are created, using a modern advanced planning technique. Inhomogeneity corrections and advanced dose calculation algorithms (Monte Carlo, Acuros, AAA, Collapsed Cone or equivalent) have to be used. The plans are optimized to keep the dose to normal tissue as low as possible and to respect the specified OAR constraints. The margin from CTV to PTV must be sufficient to ensure that 100% of the CTV volume is covered with at least 95% of the prescribed dose. These dose constraints and recommendations for dose to OARs apply regardless of randomisation to the SPECT or standard plan arm.

#### Patient setup and in-room imaging

For every treatment session, the internal target position is verified using a kV cone beam CT scan acquired in the treatment position. The patient position is corrected before treatment to ensure that the primary tumour and lymph nodes are as close to the planning situation as possible. Adaptive re-planning criteria are left up to the implemented guidelines for systematic adaptive treatment strategies.

#### SPECT/CT planning and optimisation technique

New contours of functional lung identified as 20–80% subvolumes of the maximum perfusion count (FL20–80) are used in a second optimization to avoid high radiation doses to the best functioning lung tissue. The principal objective for functional avoidance is to reduce dose to the highly perfused lung subvolumes without compromising PTV coverage and to minimise a dose variation within the PTV. Dose constraints for other OAR should be respected and kept within the predefined constraints, described above.

For each patient, two treatment plans will be generated:

1. A standard reference plan, based on CT alone, blinded to functional structures
2. A functional avoidance plan, SPECT-plan, imposing higher priority on functional levels.

Functional avoidance RT planning objectives are:

- To obtain lower (as low as possible) doses to FL volumes, while maintaining CTV/PTV coverage.
- Dose to organs at risk must be kept within the specified above dose constraints.

The standard plan is used as a basis for optimization of the functional avoidance plan.

The following mean constraints are added to keep anatomical lung and heart doses similar between the two plans. Relative tolerated change (△) in dose-volume parameters for OARs (+/−):

- total lung △mean Dose ≤1.5 Gy
- heart △mean Dose ≤1.5 Gy

Provided it still meets all normal tissue constraints described above.

Results of the feasibility study of standard and functional avoidance plans comparison between two participating centres are presented in Additional file 1.

#### Randomisation and blinding procedures

After both plans have been approved and accepted for treatment, randomisation will take place electronically. Participants will be randomly assigned to either experimental or control group with a 2:1 allocation as per a computer-generated randomisation schedule stratified by disease stage, concurrent chemotherapy, and treating institution, using permuted blocks of random sizes. The block sizes will not be disclosed, to ensure concealment. Data Manager of Clinical Research Unit will generate allocation sequence and assign participants to intervention. Allocation concealment will be ensured, as the service will not release the randomisation code until the patient has been recruited into the trial, which takes place after all baseline measurements have been completed, and two treatment plans have been approved. Randomisation will be requested from Clinical Research Unit, which will send the result to the dose planner/physicist and research coordinators who are not involved in direct patient care or study assessments. The correspondent radiation treatment plan will be sent through to the treatment Unit, while another one rejected in the treatment planning system. Information on the allocated Arm will be concealed from the treatment plan ID, and therefore concealed from radiotherapists/ radiation nurses in direct contact with the patient.

All participants and personnel involved in their medical care will be blinded to the allocated treatment arm. Research personnel, and planners/ medical physicists are unblinded. Unblinding of the treating radiation oncologist is permitted in case of serious adverse event, where treatment plan review is necessary. Unblinding of the treating radiation oncologist is also permitted in case of treatment adaptation.

#### Adaptation and re-planning

If the patient requires a repeat simulation procedure for an unforeseen reason (i.e. significant tumour reduction, atelectasis), no new SPECT/CT is performed. If the patient is in the functional avoidance arm, the baseline functional lung contours are transferred rigidly to the new planning CT, cropped to the new lung volume, and used for adaptive re-plan procedures. In case of FL volumes cannot directly be transferred and Functional Avoidance plan cannot be produced (due to large anatomical changes, atelectasis, etc), the functional volumes may be omitted in optimization. The patient may continue treatment with a standard plan. This procedure is allowed upon Specialist discretion. Site Investigator must be notified. Only the relevant treatment plan is re-optimized on the new planning CT and approved by Specialist.

### Follow-up and assessment

Participant timeline is shown in Table 1.

#### Before treatment start

All patients that meet eligibility criteria can be registered in this trial. Immediately after registration and prior to RT, baseline assessments will be made. This includes a standard physical examination, routine blood tests, SPECT/CT scan and baseline toxicity and quality of life evaluation. A baseline SPECT/CT scan will be performed < 2 weeks prior to RT. Participation in the protocol requires one extra SPECT/CT scan before initiation of RT, quality of life assessments before RT and in follow-up. Other investigations are a part of the routine clinical assessment and treatment (Table 1).

#### During treatment

The patient is seen by a physician on the day of the first, middle and last fraction, where toxicity is evaluated by CTC-AE v. 5, medication list updated, and quality of life assessed.

#### After the end of treatment (follow-up)

Follow-up visits are scheduled for 1, 3, 6, 9 and 12 months after RT. Investigations during follow-up serve the purpose of monitoring radiation-induced toxicity and quality of life. In case of lung toxicity occurrence on the scheduled follow-up visits, as well as acutely, diagnostic and treatment procedures will be done as indicated by the standard diagnostic procedures. In case of disease progression, the patient will be treated according to regular department policy.

### Statistical considerations

#### Sample size

Bases on the Quantitative Analyses of Normal Tissue Effects in the Clinic lung report and previously reported prospective trial rates [6], a freedom from RILT rate of 75% would be expected to be observed in patients receiving standard therapy. For the SPECT to be considered worthwhile for further investigation a freedom from RILT rate of at least 84% would suggest a clinically worthwhile signal in SPECT. Based on Ahern’s single arm design, a sample size of 130 patients receiving SPECT would have at least 80% power to have 95% confidence in ruling out an uninteresting freedom from RILT rate of 75% in favour of a clinically more interesting rate of 84%. In order to reduce selection bias, a randomised phase II design is proposed with a 2:1 randomisation of SPECT to standard treatment. The sample size of 195 is required and a total of 200 patients will be enrolled to offset a modest attrition rate. All patients will be followed for at least 6 months. The primary endpoint will be described by the proportion of patients in the SPECT arm are free of symptomatic RILT of at least grade 2 together with the associated 95% confidence interval. The corresponding proportion will be calculated in the control arm to provide contemporary free of symptomatic RILT of at least grade 2 rates in the standard treatment group.

It is expected that 5–6 patients per month can be accrued between the two participating countries (Denmark and Australia), thus, it is expected that accrual to this study could be completed in 3 years.

#### Statistical analysis

Analyses will be based on the principle of intention-to-treat. Descriptive statistics will be used to summarize patient characteristics and endpoints separately in each of the treatment groups. Estimates in each group will be reported by percentage experiencing RITL together with the corresponding 95%CI.

Exploratory analyses investigating association of clinical/patient/planning factors on outcomes will be performed using standard statistical methods (t-test, multivariate regression methods, and chi-squared tests). Time-to-event outcomes will be described using the method of Kaplan-Meier and any exploratory comparisons using logrank tests and proportional hazards regression. No imputation will be made for missing data. Estimation of the relationship between DVH parameters and toxicity will be evaluated using regression methods and correlations between functional and conventional DVH parameters will be evaluated. All patients who are randomised will be included in the analysis of safety and efficacy outcomes. Any patient who is randomised but does not receive radiotherapy based on the intervention allocated treatment plan will be described in detail along with the reason for not receiving the prescribed treatment.

#### Data safety monitoring committee and interim analysis

The data safety monitoring committee will consist of a statistician, an independent investigator, and a data manager from Clinical Trial Unit. The committee will review serious adverse events on annual basis. An interim analysis for futility is proposed to be performed after 50 patients randomised to SPECT have completed treatment and have been followed for at least 6 months. The futility boundary is determined from a likelihood ratio approach which suggest that if the ‘support’ for the interesting rate based on the likelihood ratio (84% rate: 75% rate) is LR ≤ 0.125 then the study could be considered futile in its ability to yield an estimate consistent with a freedom from RILT of at least 84%. For 50 patients, fewer than 35 patients are free from RILT, the LR ≤ 0.065. If this is observed, consideration will be given to either (i) modify the protocol (inappropriate control rate); (ii) alter the study design or (iii) stop the study. An independent Data Safety and Monitoring Committee will oversee the safety of the study as well as review the results of the 50-patient futility analysis. Otherwise, the study would proceed to recruit an additional 80 patients in Arm 1. Arm 2 would recruit in all 65 patients.

#### Ethics approval

This protocol and the template informed consent forms have been reviewed and approved by the sponsor and the applicable ethical committee of each participating research centre with respect to scientific content and compliance with applicable research and human subjects’ regulations. The responsible investigator will ensure that this study is conducted in agreement with the Declaration of Helsinki. Written informed consent will be obtained from all patients for the acquisition and use of anonymized clinical data before they are recruited. All personal health information will be kept strictly confidential. All participants will be identified using initials and a unique identification number. Confidential subject identification list will be kept at Clinical Trial Unit. Trial Management Committee will oversee the intra-study data sharing process. All lead Investigators will be given access to the cleaned data sets. Project data sets will be password protected. Principal investigator will have direct access to all site’s data sets. To ensure confidentiality, data dispersed to project team members will be blinded of any identifying participant information. Published data will not contain any identification information of participants. Randomised controlled trial shall be presented according to the CONSORT guidelines. Regardless the outcome of the trial, positive, negative, and inconclusive results will be published. Preliminary results are expected after interim analysis. Authorship of the trial abstract and manuscript will be decided by the principal investigator at the time of submission. The Investigator will make safety and progress reports to the Ethics Committee annually and within 3 months of study termination or completion at their site. These reports will include the total number of participants enrolled and summaries of each safety and efficacy review.

## Discussion

Over the last two decades, various functional modality techniques have been investigated for use in lung cancer RT, such as perfusion/ventilation SPECT, 4DCT (ventilation), hyperpolarized ^3^He Magnetic resonance imaging (MRI) (ventilation), Gallium-PET (perfusion/ventilation) and hyperpolarized ^129^Xe MRI [11, 13–15]. As lung function consists of three components- airways ventilation, gas exchange in alveoli and perfusion of blood vessels, ideally, combined imaging of perfusion and ventilation, will provide the most accurate imaging of pulmonary function. Never-the-less for RT planning purposes, perfusion imaging appears to be sufficient [16–18]. Indeed, perfusion imaging is the most commonly used form of functional lung imaging reported in the literature [8]. In regard to the lung function assessment for RT of lung cancer and other types of cancers involving thoracic irradiation (e.g. breast cancer, cancer in chest wall, lymphomas), perfusion has shown a predictive potential for radiation-induced toxicity in a consistent dose-response relation [19–24]. Considering our previous expertise in establishing the role of SPECT in radiation-induced lung injury, and multi-centre experience incorporating functional avoidance into RT planning, SPECT/CT imaging was found to be most feasible and pragmatic functional imaging for the ASPECT trial.

The results of the inter-institutional comparability planning study show that standard and functional avoidance plans are comparable between the centres (Additional file 1). Dose planning objectives were met in 4 out of 5 patients. Both centres performed standard planning with minimal difference in PTV coverage. Some differences were observed in doses to total lung and heart. Functional planning objective of minimizing dose to the functional lung without significant dose increase to organs at risk was achieved for 4 out of 5 patients. For one patient standard dose planning constraints, as well as functional objectives were not achieved at one centre due to large target volume (PTV volume 616 cm^3^). Differences between Centre 1 and Centre 2 standard and functional plans (∆ Standard and ∆ Functional) were acceptable. Dose reduction to FL, achieved by functional avoidance plans for Centre 1 and Centre 2 were comparable. As shown in Supplementary Table S1, dose reduction to FL was comparable to the previously published results [8, 12].

The ASPECT trial will be the first randomised clinical trial incorporating SPECT/CT functional imaging into radiotherapy of lung cancer assessing the clinical outcomes. The project will be of great international significance, as methods to improve the treatment outcome of lung cancer patients are warranted worldwide. If successful, the trial has a potential to improve the treatment of lung cancer and broaden the category of patients receiving radiotherapy. The trial can potentially lead to tumour dose intensification and further improve patients’ prognosis.

In summary, ASPECT trial will investigate functional avoidance method of RT in clinical practice, and will establish toxicity outcomes for patients with lung cancer undergoing curative chemo-RT.

## Supplementary Information

**Additional file 1.**

## Data Availability

All data generated or analysed during this study are included in this published article and its supplementary files. After finishing the enrollment and follow-up of the participants, the raw datasets are available from the corresponding author on reasonable request.

## Abbreviations

ASPECT: Avoidance SPECT-guided
CTV: Clinical target volume
DLCO: Diffusing capacity of the lungs for carbon monoxide
DOLG: Danish Oncologic Lung Cancer Group
DVH: Dose-volume histogram
EORTC: European Organisation for Research and Treatment of Cancer
FL: Functional lung
GTV: Gross tumour volume
MLD: Mean lung dose
MRI: Magnetic resonance imaging
NCI-CTC: National Cancer Institute Common Toxicity Criteria
OAR: Organs at risk
QLQ: Quality of life questionnaire
QOL: Quality of life
PD-L1: Programmed death ligand-1
PET: Positron emission tomography
PFT: Pulmonary function tests
PTV: Planning target volume
RILT: Radiation-induced lung toxicity
RT: Radiation therapy
SPECT: Single photon emission computed tomography
