# Supplementary material for: Personal innovative approach in radiation therapy of lung cancer- functional lung avoidance SPECT-guided (ASPECT) radiation therapy: a study protocol for phase II randomised double-blind clinical trial
Source: BMC Cancer. 2021 Aug 21;21:940. doi: 10.1186/s12885-021-08663-1 (PMC8379730; doi:10.1186/s12885-021-08663-1)
Supplement: Supplementary file 1 — Additional file 1. [file 12885_2021_8663_MOESM1_ESM.docx]

Additional File 1. Radiation treatment plan comparison study between participating centres.

The aim of the study was to create radiation treatment plans for five selected patients at two collaborating centres- Aarhus University Hospital (Centre 1) and Westmead Hospital, Sydney West Radiation Oncology Network (Centre 2), and to compare standard and functional dose-volume parameters between the centres.

In all five patients were selected from the previous study [9]. Selection was based on the size of target volume and mean lung dose on the given radiation treatment plan. Planning CT scans with target volumes and organs at risk were de-identified, and used for planning at Centre 1 and 2. Two plans were created for each patient- standard, based on CT, and functional considering functional lung (FL 20-80%) as organ at risk [10]. Dose plan objectives for standard plan:

Target dose and coverage: 66 Gy in 33 fractions; Planning treatment volume (PTV)  V95% > 99%  CTV  V95% = 100% 
Spinal Cord  D0.05cm3 < 45Gy 
PRV Spinal Cord  D0.05cm3 < 50Gy 
Lung Total  MLD < 19Gy, V20 < 35%, V5Gy < 60% 
Esophagus  D1cm3 < 66Gy 
Heart  V40Gy < 30% , V25Gy < 50% 
Hot spots: Body  D0.05cm3 < 110%, Body  D5cm3 < 107% .

Planning objective for functional plan was to optimize plans according to FL 20-80% volumes distribution, in order to minimise dose to FL 20-80% in ascending priority. Dose to CT-derived total lung volume was to remain within 1.5 Gy of difference between standard and functional plans.

Following parameters were derived from each plan at Centre 1 and 2 and compared:

- PTV and coverage of 95% of PTV
- Volume and mean dose to functional lung volumes (FL 20-80%) from SPECT/CT
- Volume, mean dose and Vx total lung from CT alone
- Dose and Vx for heart and esophagus

Results for each individual patient’s standard and functional plans from Centre 1 and Centre 2 are presented in Supplementary Table S1.

|  | **Centre 1** | |  | |  | |  | |  | | |  |  | |  | |  | |  | |  | | |  | **Centre 2** | |  | |  | |  | |  | | |  |  | |  | |  | |  | |  | | |  | **∆ C1-C2*** | |  | |
| --- | --- | --- | --- | --- | --- | --- | --- | --- | --- | --- | --- | --- | --- | --- | --- | --- | --- | --- | --- | --- | --- | --- | --- | --- | --- | --- | --- | --- | --- | --- | --- | --- | --- | --- | --- | --- | --- | --- | --- | --- | --- | --- | --- | --- | --- | --- | --- | --- | --- | --- | --- | --- |
|  | Standard dose plan | | | | |  | |  | |  | Functional avoidance dose plan | | | | | | | | |  | |  | Standard dose plan | | | | | | |  | |  | |  | Functional avoidance dose plan | | | | | | | | |  | |  | ***∆Standard*** | | | ***∆Functional*** | |  |
| **Patient nr** | 1 | 2 | | 3 | | 4 | | 5 | |  | 1 | | | 2 | | 3 | | 4 | | 5 | |  | 1 | | | 2 | | 3 | | 4 | | 5 | |  | 1 | | | 2 | | 3 | | 4 | | 5 | |  |  | | |  | |  |
| **Parameter:** |  |  | |  | |  | |  | |  |  | | |  | |  | |  | |  | |  |  | | |  | |  | |  | |  | |  |  | | |  | |  | |  | |  | |  |  | | |  | |  |
| PTV V95 | 99,27 | 99,20 | | 99,02 | | 99,09 | | 99,00 | |  | 99,13 | | | 99,13 | | 99,37 | | 99,22 | | 98,36 | |  | 100,00 | | | 99,19 | | 99,97 | | 99,84 | | 99,83 | |  | 99,99 | | | 99,07 | | 99,98 | | 99,96 | | 99,85 | |  | -0,7 | | | -0,7 | |  |
| Mean dose to FL40, Gy | 11,04 | 14,62 | | 10,91 | | 6,85 | | 17,00 | |  | 10,22 | | | 13,91 | | 8,76 | | 5,99 | | 15,50 | |  | 16,27 | | | 17,70 | | 11,82 | | 6,29 | | 26,87 | |  | 14,85 | | | 16,21 | | 7,42 | | 5,40 | | 28,16 | |  | 3,7 | | | 3,5 | |  |
| Mean lung dose, Gy | 13,86 | 14,22 | | 11,52 | | 12,88 | | 15,76 | |  | 14,12 | | | 13,87 | | 11,48 | | 12,64 | | 14,91 | |  | 18,43 | | | 17,54 | | 13,20 | | 13,48 | | 23,42 | |  | 17,83 | | | 16,40 | | 11,97 | | 13,13 | | 24,16 | |  | 3,6 | | | 3,3 | |  |
| Lung V5, % | 36,95 | 46,50 | | 45,85 | | 53,66 | | 59,87 | |  | 40,56 | | | 45,00 | | 40,45 | | 50,10 | | 58,05 | |  | 64,68 | | | 57,36 | | 62,10 | | 42,49 | | 85,15 | |  | 62,91 | | | 54,19 | | 38,36 | | 38,33 | | 86,78 | |  | 13,8 | | | 9,3 | |  |
| Lung V20, % | 22,69 | 24,48 | | 17,85 | | 21,18 | | 28,36 | |  | 22,18 | | | 23,66 | | 19,31 | | 21,75 | | 25,38 | |  | 28,17 | | | 32,09 | | 17,12 | | 23,40 | | 46,82 | |  | 27,29 | | | 31,83 | | 20,72 | | 23,31 | | 46,65 | |  | 6,6 | | | 7,5 | |  |
| Heart V40, % | 0,00 | 4,14 | | 7,01 | | 3,11 | | 11,52 | |  | 0,00 | | | 5,27 | | 7,42 | | 3,26 | | 10,84 | |  | 0,08 | | | 21,53 | | 16,97 | | 7,19 | | 26,91 | |  | 0,03 | | | 20,96 | | 14,31 | | 16,03 | | 27,88 | |  | 9,4 | | | 10,5 | |  |
| Heart V25, % | 0,11 | 7,82 | | 11,61 | | 7,54 | | 22,78 | |  | 0,00 | | | 9,28 | | 13,31 | | 7,91 | | 21,92 | |  | 1,08 | | | 37,67 | | 36,24 | | 43,20 | | 49,94 | |  | 0,49 | | | 44,22 | | 30,55 | | 44,26 | | 50,61 | |  | 23,7 | | | 23,5 | |  |
| Esophagus dose max, Gy | 59,00 | 68,56 | | 35,69 | | 67,33 | | 67,66 | |  | 63,73 | | | 68,76 | | 31,97 | | 67,67 | | 67,41 | |  | 65,75 | | | 69,79 | | 10,34 | | 68,30 | | 69,12 | |  | 65,25 | | | 69,58 | | 22,31 | | 66,77 | | 68,58 | |  | -3,0 | | | -1,4 | |  |
| Esophagus dose to 1cc, Gy | 42,11 | 65,68 | | 28,18 | | 64,97 | | 65,34 | |  | 57,90 | | | 65,41 | | 20,73 | | 65,37 | | 65,62 | |  | 58,44 | | | 63,83 | | 32,73 | | 66,89 | | 66,71 | |  | 57,13 | | | 65,16 | | 17,11 | | 66,15 | | 67,42 | |  | 4,5 | | | -0,4 | |  |
| *mean difference between Centre 1 and Centre 2 | | | |  | |  | |  | |  |  | | |  | |  | |  | |  | |  |  | | |  | |  | |  | |  | |  |  | | |  | |  | |  | |  | |  |  | | |  | |  |

|  |  | **Centre 1** | **Centre 2** |
| --- | --- | --- | --- |
| Mean dose reduction to | FL20%, Gy | 0,35 | 0,80 |
|  | FL40%, Gy | 1,21 | 1,39 |
|  | FL60%, Gy | 3,17 | 1,80 |
|  | FL80%, Gy | 8,25 | 3,27 |

Supplementary Table S1. Results for individual patients’ dose plans, standard and functional avoidance, performed at two centres.
